# Inferior Wall ST-Elevation Myocardial Infarction Complicated by Left Ventricular Pseudoaneurysm, Cardiac Tamponade, and Partial Papillary Muscle Rupture

**DOI:** 10.7759/cureus.24061

**Published:** 2022-04-12

**Authors:** Michel El Khoury, Samer Saouma, David Ayad, Nnedi Asogwa, Harout Yacoub

**Affiliations:** 1 Internal Medicine, Staten Island University Hospital, Northwell Heath, Staten Island, USA; 2 Cardiology, Staten Island University Hospital, Northwell Heath, Staten Island, USA

**Keywords:** mitral valve, mitraclip, transthoracic echocardiography, myocardial infarction, papillary muscle rupture

## Abstract

Papillary muscle rupture (PMR) is a rare complication of myocardial infarction. Its incidence has been decreasing nowadays because of improved early revascularization techniques. When it occurs, surgical treatment is the only therapeutic lifesaving approach. We report a case of an 85-year-old female patient who presented to the emergency room with chest pain. An electrocardiogram showed inferior wall ST-elevation myocardial infarction. The patient was revascularized emergently with a drug-eluting stent to the obtuse marginal artery. An intra-aortic balloon pump was inserted for hemodynamic support. Six hours later, the patient developed shortness of breath with persistent hypotension. A transthoracic echocardiogram (TTE) showed a large pericardial effusion with a pseudoaneurysm in the infero-septal apex. Immediate drainage of pericardial fluid was performed. Seventy-two hours later, the patient had flash pulmonary edema. A new severe eccentric mitral regurgitation was discovered on transesophageal echocardiography (TEE). Findings revealed a partial posteromedial papillary muscle tear and prolapse of the A2 scallop. The patient was not a candidate for surgical replacement or percutaneous repair due to the high surgical risk and poor functional status, and she passed away on day fifteen of her hospital stay. Limited case series have shown promising benefits of percutaneous edge-to-edge mitral valve repair in selected high surgical risk patients and as a bridge to definitive mitral valve replacement. A diagnosis of PMR should be in the differential diagnosis, especially when evaluating hemodynamically unstable patients who present with prolonged symptoms.

## Introduction

Papillary muscle rupture (PMR) usually happens four to five days post-myocardial infarction (MI) and occurs in 0.1% of patients in view of advanced reperfusion techniques [1]. This muscle rupture can lead to acute severe mitral regurgitation (MR) resulting in left ventricular (LV) failure, cardiogenic shock, and florid pulmonary edema. Because its blood supply is derived solely from the posterior descending artery, rupture of the posteromedial papillary muscle occurs three to 12 times more frequently than that of the anterolateral counterpart [2]. The right coronary artery is often the culprit lesion [3]. The treatment is usually with surgical repair or replacement, but it carries a substantial perioperative morbidity and mortality rate. We herein report a case of an elderly patient who presented to the emergency room with MI and developed multiple mechanical complications including partial PMR, cardiac tamponade, and LV pseudoaneurysm.

## Case presentation

**Figure 1:**
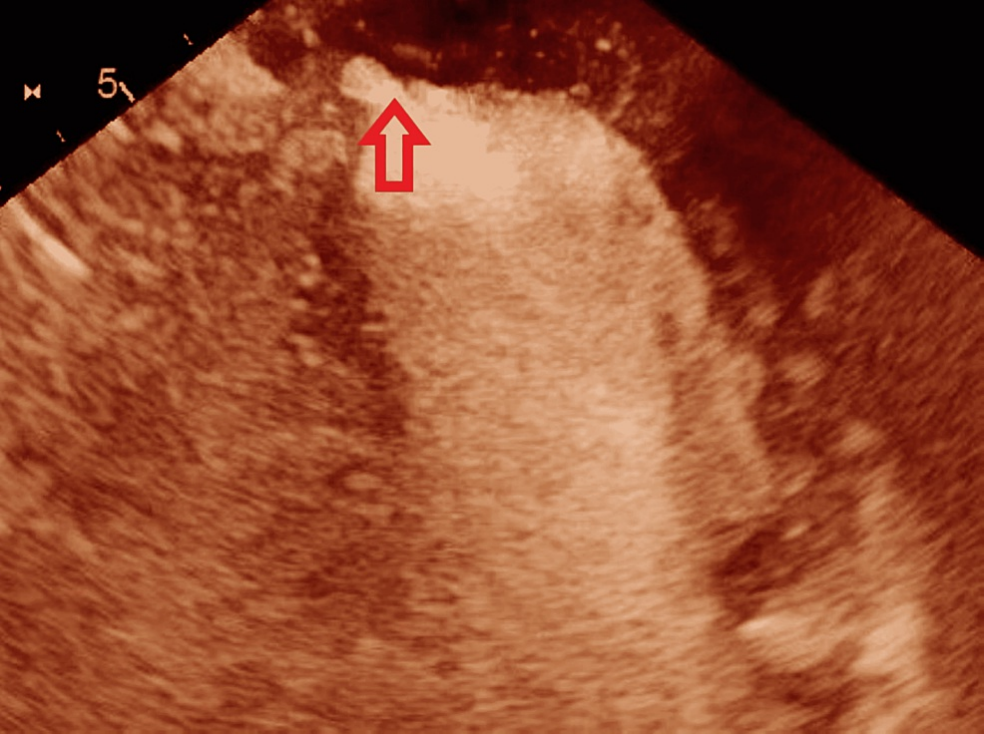
Echocardiography with contrast shows left ventricular pseudoaneurysm

An 85-year-old female patient presented to the emergency room with three weeks history of shortness of breath and chest pain. Past medical history is significant for ischemic heart disease status post two stents insertion, hypertension, and carotid artery stenosis. Past surgical history is significant for cataract surgery. She is a non-smoker and denies any illicit drug use. She had a normal cardiopulmonary exam and vital signs. Electrocardiogram showed inferior wall MI and the patient underwent emergent cardiac catheterization with percutaneous coronary intervention to the first obtuse marginal artery with drug-eluting stent placement. Coronary angiography showed triple vessel disease with a SYNTAX I score of 15. The coronary circulation was right dominant. She was started on aspirin and ticagrelor. During catheterization, the patient developed acute pulmonary edema and cardiogenic shock requiring intra-aortic balloon pump insertion for hemodynamic support. Six hours later, she remained hypotensive despite dual vasopressor therapy. Echocardiography revealed cardiac tamponade. Successful emergent pericardiocentesis was performed. Follow-up echocardiography was performed, and it revealed LV apical pseudoaneurysm, an ejection fraction of 45%, grade 2 diastolic dysfunction, severely enlarged left atrium, anterior mitral leaflet prolapse, and severe eccentric mitral regurgitation (Figure 1). A decision was made to proceed with transesophageal echocardiography (TEE) to assess the valve morphology and suitability for a percutaneous edge-to-edge mitral valve repair.

Imaging findings

**Figure 2:**
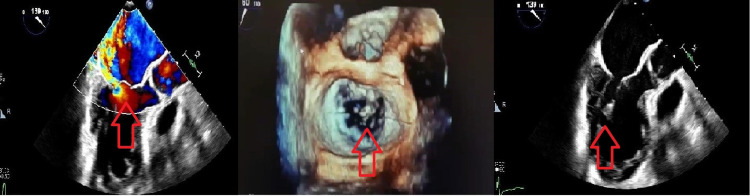
Flail anterior leaflet due to posteromedial papillary muscle rupture with a posteriorly direct jet

Emergent TEE revealed a partial posteromedial papillary muscle tear and prolapse of the A2 scallop. Mitral regurgitation effective regurgitant orifice was 0.58 cm^2^ and regurgitant volume 62 ml (Figure 2).

Role of imaging in patient care

With a sensitivity of 65-85%, echocardiography can help diagnose papillary muscle rupture. On echocardiography, prolapse or flail leaflet, as well as the direction of the regurgitant jet, may be easy to detect, but these signs do not consistently predict the affected papillary muscle, and direct sight of the rupture is required for final diagnosis [4]. It's important to remember that in cases of partial PMR, a transthoracic echocardiography examination can be nondiagnostic, while transesophageal echocardiography has a high diagnostic sensitivity.

The patient was deemed not a candidate for percutaneous edge-to-edge mitral valve repair or surgical intervention. Given her unstable condition, a decision was made for palliative extubation, and the patient passed away on day 15 of the hospital stay.

## Discussion

Mechanical complications of acute myocardial infarction include rupture of the left ventricular free wall, rupture of the interventricular septum, pseudoaneurysm, and papillary muscle rupture [5]. This could lead to hypotension, pulmonary congestion, and multiorgan failure resulting in death within hours of diagnosis if these conditions are left untreated. In our patient, cardiac tamponade complicated by LV pseudoaneurysm, and partial PMR were diagnosed. She was not a candidate for surgical or percutaneous mitral valve repair and unfortunately, she passed away few days after her admission. Risk factors for PMR include older age, female sex, a history of heart failure, chronic kidney disease, and a delayed presentation [5]. The necessity of understanding mitral valve anatomy is shown in this situation. An anterior and posterior leaflet make up the valve itself. The two papillary muscles (anterolateral and posteromedial) that are linked to the leaflets via the chordae tendinae maintain valvular competence. The anterolateral muscle is often a single big component, whereas the posteromedial muscle has one to three heads [6]. Because both papillary muscles supply chordae tendinae to both mitral valve leaflets, a rupture of either papillary muscle can harm either leaflet. The left anterior descending and circumflex arteries both send blood to the anterolateral muscle, but the posteromedial muscle is supplied solely by the right coronary artery. Thus, the posteromedial muscle is more prone to rupture.

Treatment strategies in patients with post-MI PMR are sparse. According to Bouma et al., in-hospital mortality in patients with complete PMR was 42%, while it was more than three times lower in patients with partial PMR [7]. Emergent surgical replacement is considered the only option available for treating this condition [8], however, most of these patients carry substantial perioperative mortality [9]. A recent analysis of acute MI admissions from the National Inpatient Sample found that only 58% of patients with PMR underwent mitral valve surgery [10]. On the other hand, if acute MR is the result of partial PMR, and the extent of damage to adjacent myocardial tissues is limited, repair of the mitral apparatus may be considered [11,12].

A new article has been published by Budra et al. reporting successful treatment with rescue transventricular off-pump mitral valve repair with artificial neochords in three patients diagnosed to have post-MI PMR [13]. This case series is limited by its retrospective descriptive design and small sample size, as are other reports on PMR in the literature. This technique may stabilize high-risk patients with acute MR and cardiogenic shock and serve as a bridge to conventional surgery. Once the patient becomes a stable surgical candidate with lower operative risk, a conventional approach with surgical replacement must be attempted. In the modern era of evolving minimally invasive surgical techniques, the use of MitraClip (Santa Clara, CA: Abbott Vascular) has been tried in acute ischemic MR without papillary muscle rupture. This was proven by Estevez-Loureiro et al. [14]. In fact, the MitraClip device was found to be possible in selected individuals with acute ischemic MR, with an 86.6% success rate and a median of two clips per case. 

In addition to that, several isolated case reports of post-MI PMR that were successfully treated with the MitraClip device were found in a literature review [15-22]. Additional studies are necessary to establish the long-term efficacy and safety of MitraClip use in this catastrophic complication. Medical therapy as a bridge to candidacy for mitral valve replacement, and temporary mechanical assistance as a bridge to long-term ventricular assist device or heart transplantation are alternate therapeutic options for individuals with contraindications to surgical mitral valve repair.

## Conclusions

Our case represents a rare dreadful complication of acute MI. Surgery is usually the preferred treatment, but it is often postponed due to high surgical mortality. Fortunately, as the use of early and successful revascularization therapy has increased, PMR has become a very uncommon consequence. However, clinicians should be aware of this potentially fatal consequence and be ready to diagnose it quickly. especially in patients presenting with prolonged symptoms like our case. New percutaneous interventions, including MitraClip implantation, have been tried in small case series and have resulted in a lower mortality rate. Because of publication bias and selective reporting of successful operations, caution should be exercised when interpreting outcomes from existing literature. The decision should be made by a multidisciplinary team of experts in structural heart disease and cardiothoracic surgery based on risk assessment scores, clinical presentation, mitral valve morphology, and cardiac function.
